# Natriuretic Hormones in Brain Function

**DOI:** 10.3389/fendo.2014.00201

**Published:** 2014-11-28

**Authors:** Anastasia Hodes, David Lichtstein

**Affiliations:** ^1^Faculty of Medicine, Department of Medical Neurobiology, Institute for Medical Research Israel-Canada, The Hebrew University of Jerusalem, Jerusalem, Israel

**Keywords:** atrial natriuretic peptide, cardiac steroids, ouabain, guanylin, brain function

## Abstract

Natriuretic hormones (NH) include three groups of compounds: the natriuretic peptides (ANP, BNP and CNP), the gastrointestinal peptides (guanylin and uroguanylin), and endogenous cardiac steroids. These substances induce the kidney to excrete sodium and therefore participate in the regulation of sodium and water homeostasis, blood volume, and blood pressure (BP). In addition to their peripheral functions, these hormones act as neurotransmitters or neuromodulators in the brain. In this review, the established information on the biosynthesis, release and function of NH is discussed, with particular focus on their role in brain function. The available literature on the expression patterns of each of the NH and their receptors in the brain is summarized, followed by the evidence for their roles in modulating brain function. Although numerous open questions exist regarding this issue, the available data support the notion that NH participate in the central regulation of BP, neuroprotection, satiety, and various psychiatric conditions, including anxiety, addiction, and depressive disorders. In addition, the interactions between the different NH in the periphery and the brain are discussed.

## Introduction

Natriuretic hormones (NH) are compounds that act in an endocrine or paracrine fashion to regulate extracellular fluid volume and blood pressure (BP) through the stimulation of sodium excretion by the kidney. Three groups of compounds fall into this broad definition: the natriuretic peptides (NP: ANP, BNP, and CNP), the guanylin peptides (GP), and the endogenous cardiac steroids (CS: ouabain, digoxin, and marinobufagenin). A large body of evidence supports the notion that in addition to their natriuretic effects, these hormones participate in numerous brain functions. Our goal is to review the established information on the biosynthesis, release, and physiological roles of NH, with particular focus on the brain. The available literature on the interactions between the different NH families in the periphery and in the brain is also addressed.

## Natriuretic Peptides

The first demonstration of an endocrine link between the heart and kidneys came from the pioneering experiments of De Bold, which led to the discovery of atrial NP (ANP), the founding member of the family of NP. De Bold and his colleagues found that injecting rats with an atrial homogenate caused significant natriuresis and diuresis (1). Additional members of this family of peptides were purified over the course of the following years: B-type NP (BNP) (2) and C-type NP (CNP) (3). ANP, BNP, and CNP are expressed as pre-pro-hormones and are proteolytically processed to form the mature peptides. The three peptides share a similar structure consisting of two cysteine residues flanking a 17-residue disulfide-linked ring that is essential for biological activity (3). The main inducer of ANP release is atrial wall stretch (4). BNP is released from the atrium, as is ANP, but its main sources are the ventricles, where BNP is transcriptionally regulated by cardiac wall stretch resulting from volume overload (5). There are three known NP receptors: NP receptor-A (NPR-A), or guanylyl cyclase A (GC-A), which binds ANP and BNP (6); NPR-B (GC-B), which is highly specific for CNP (7); and NPR-C. NPR-A and NPR-B are membrane-bound receptors consisting of an extracellular ligand binding domain, a single transmembrane region and an intracellular GC domain that rapidly releases cyclic guanosine monophosphate (cGMP) in response to the NP binding (8). The cGMP then acts as a second messenger that activates protein kinase-G (PKG) and subsequent cellular signaling cascades (9). A third receptor, NPR-C, contains only a short intracellular fragment and has no GC activity. The main function of NPR-C is to clear NP through receptor-mediated internalization and degradation (10).

### Physiological roles of NP

Atrial natriuretic peptide has a major role in the regulation of BP. In the kidney, ANP induces natriuresis and diuresis by increasing the glomerular filtration rate (GFR) and inhibiting sodium and water reabsorption (11). ANP acts as a functional antagonist of the renin–angiotensin–aldosteron system by inhibiting renin secretion from the kidney and aldosterone production in the adrenal glands (12). It stimulates smooth muscle cell relaxation in blood vessels, causing vasorelaxation (13). It also regulates the intravascular volume by increasing endothelium permeability (14). In accordance with these effects, it was found that ANP knockout mice developed salt-sensitive hypertension (15). ANP also directly affects the heart by inhibiting cardiac hypertrophy (16). BNP activates the same receptor as ANP but its precise functional significance is not well understood. Studies on mice with targeted disruption of BNP (17) and on cultured cardiac fibroblasts (18) established BNP as an antifibrotic factor that plays a role in ventricular remodeling. Indeed, high concentrations of BNP were found in the ventricles following congestive heart failure or myocardial infarction, rendering it an important biomarker for these conditions (19). Unlike the other two family members, CNP acts in an autocrine/paracrine fashion. Although NPR-B is present in the kidney, CNP has little natriuretic or diuretic effect, and it is a much more potent cardiovascular effector (20). CNP is produced by the endothelium and induces vasorelaxation (21). It also participates in vascular remodeling following injury (22). In addition to their cardiovascular and renal effects, NP show a wide-spread effect throughout the body (8): They act as bronchodilators and vasorelaxants in the lungs (23), elicit anti-inflammatory effects in the immune system (24), and have metabolic effects on the adipose tissue (25) and on long bones (26).

### NP in the brain

The ANP, BNP, and CNP and their receptors are expressed in the brain, which implies a possible role for these peptides in brain function. CNP is the most abundantly present NP in the brain (27), suggesting that it acts as a neurotransmitter or neuromodulator rather than a cardiac hormone (28). Accordingly, the CNP-specific receptor – NPR-B is widely spread throughout the brain: NPR-B mRNA was detected in the cerebral cortex, the limbic area, preoptic-hypothalamic regions, motor nuclei, and the brainstem (29). ANP and BNP are also present in the brain and have interesting neuromodulatory functions. ANP expression was first found in the hypothalamus (30), which is the main source of NP in the brain (31, 32). ANP is present in neurons and glia in the cerebral cortex (33) and in the cerebellum (34). ANP was also described in neurons and fibers in the limbic area, olfactory bulb, thalamus, and striatum (31, 35–37). BNP was found in the hypothalamus (38) and cerebral cortex (33). Unlike ANP and CNP, no BNP mRNA was detected in the brain (39), suggesting the peripheral origin of this peptide. Interestingly, ANP and BNP were described in some of the circumventricular organs in the brain – the highly vasculated structures in the hypothalamus that allow endocrine communication between the periphery and CNS (40). Considerable ANP-like immunoreactivity was found in nerve fibers of the vascular organ of the lamina terminalis and the subfornical organ in rat brain (31). Neurons in the subfornical organ were shown to respond to ANP by increased cGMP production (41). Neurons of the circumventricular organs express receptors for the majority of the cardiovascular hormones (42), including NP receptors: NPR-A and NPR-B were found in the vascular organ of lamina terminalis, the subfornical organ, area postrema, and the choroid plexus (43).

#### NP in central regulation of BP

The presence of NP and their receptors in the brain, and in the circumventricular organs in particular, led to the postulation that NP, either locally produced in the brain or arriving via the peripheral circulation, might affect neuronal pathways that centrally regulate BP. However, the results are inconsistent. Intracerebroventricular (i.c.v.) administration of ANP was reported to cause a decrease in BP in normal and spontaneously hypertensive conscious rats, but only at concentrations 10 times higher than the physiological level (44). Low concentrations of ANP were shown to have a depressor effect in anesthetized rats with sinoartic denervation, leading to a decrease in BP and sympathetic outflow (45, 46). A study performed on conscious sheep showed that CNP, but not ANP, decreased BP upon i.c.v. administration (47). Numerous studies found no change in BP upon central administration of ANP (48–52) or BNP (53). However, there are reports describing a decrease in vasopressin secretion following central ANP infusion, suggesting that ANP and vasopressin may interact to attenuate the central pressor effects of vasopressin (49, 51–54). Pretreatment of rats with i.c.v. BNP was also shown to suppress vasopressin secretion (53). In several studies it was postulated that ANP acts in the brain by partially inhibiting the angiotensin II (ANG II) pathway. ANP injection prevented the pressor effect of centrally administered ANG II (46, 51). On the behavioral level, centrally administered ANP was shown to inhibit water intake induced by ANG II or dehydration in rats (55). It was also found to attenuate salt appetite in spontaneously hypertensive rats (SHR) (48). These results suggest that ANP may not be directly involved in central regulation of BP, but rather act as a secondary modulator of other mechanisms, perhaps, similar to its peripheral effect, by counteracting to the effects of vasopressin and ANG II.

#### NP in neuroprotection

Natriuretic peptides were shown to exert a neuroprotective effect in cultured cells and *in vivo*. Cortical spreading depression (CSD) is a wave of depolarization followed by transient suppression of electrical activity in the brain (56). Rats preconditioned with an evoked episode of CSD were protected from neuronal damage following cerebral ischemia (57). Wiggins and his colleagues found that an acute episode of CSD caused an elevation in ANP mRNA and peptide levels in the rat cortex. The elevation was prolonged, overlapped the time window for CSD-induced neuroprotection and accompanied by ANP-dependent activation of cGMP signaling cascades (58). Increased cGMP levels were previously implicated in the neuroprotective mechanism of CSD (59). This notion is supported by studies showing that ANP and BNP caused an elevation in cGMP levels and inhibited apoptosis of PC12 cells (60). However, there is no direct evidence of this effect in the brain. A neuroprotective effect was also demonstrated in rat retinal neurons, where ANP was shown to ameliorate NMDA-induced neurotoxicity, presumably in a dopamine-dependent manner (61). It was postulated that the ANP neuroprotective effect is mediated via the cerebral blood flow. Indeed, an increased number of ANP-immunoreactive astrocytes and other glial cells were found in the white matter surrounding an infarction area in rats (62). This neuroprotective effect may be modulated by cGMP signaling, since cGMP-phosphodiesterase inhibitor was found to have a protective effect in a focal brain injury model in rats (63). In ischemic brain edema induced in rats, intravenous (i.v.) administration of ANP proved to have a beneficial effect. At pharmacological doses, the peptide significantly suppressed the elevation of the brain’s water and sodium content and reduced the area of edema, as revealed by magnetic resonance imaging (MRI) (64). ANP was beneficial even after delayed administration, and reduced brain edema when injected i.c.v. 4 h after induction of hemorrhagic brain injury in rats (65). BNP too was implicated in neuroprotection following brain injury. James and colleagues demonstrated that i.v. administration of BNP improved cerebral blood flow and reduced inflammation in brain injury models in mice, as manifested by reduced neurodegeneration and improved functional outcome (66). Although these experiments were performed using high doses of exogenous human recombinant BNP (nesiritide), endogenous BNP may play a role in recovery from brain injury, as elevated BNP levels have been associated with this condition. Elevated plasma BNP levels were described in patients with traumatic brain injury (67, 68), stroke (69), and other brain injuries (70, 71). Elevated BNP levels were also reported in the cerebrospinal fluid (CSF) of brain trauma patients (67). These changes, however, correlated with a poor clinical outcome in trauma and stroke patients (72, 73). This may indicate an insufficiency of the endogenous neuroprotective mechanism. The mechanism of NP neuroprotection could be mediated through immunomodulation, as was demonstrated in the periphery (74). All these clinical and pre-clinical observations lead to the premise that ANP and BNP are part of an endogenous protective mechanism in the brain against injury or damage.

#### NP in behavior

Natriuretic peptides modulate the function of the hypothalamic–pituitary–adrenal (HPA) axis and influence anxiety and addictive behavior. NP regulate the HPA-axis at several levels: ANP inhibits the release of corticotrophin (ACTH) and corticortrophin releasing hormone (CRH) (75, 76), which, in turn, stimulate ANP release, acting in a feedback loop (76). ANP also directly inhibits cortisol secretion, whereas CNP exerts the opposite effect (77). Central or peripheral administration of ANP decreased anxiety-associated behavior in rats (78). In humans, lower levels of ANP were described in patients with anxiety-related disorders, including panic disorder (79) and posttraumatic stress disorder (80), and high ANP levels were associated with lower anxiety levels in patients recovering from cardiac failure (81). Experimentally induced panic attacks were followed by an increase in plasma ANP levels, which was faster and more pronounced in panic disorder patients (79, 82). These observations suggest a therapeutic potential for ANP agonists in the treatment of anxiety-related disorders (83). Indeed, pretreatment with i.v. ANP significantly reduced the number of experimentally induced panic attacks in panic disorder patients and in healthy individuals (84, 85). The effects of ANP on anxiety are presumed to be mediated through inhibition of the HPA-axis. In healthy individuals, pretreatment with ANP was able to partially block the sympathetic activation induced by a bolus injection of CRH (86). However, further investigation is needed to fully understand the interplay between ANP and the HPA-axis.

B-type natriuretic peptide, like ANP, was found to have an anxiolytic effect (87). On the other hand, CNP enhances cortisol secretion (77) and has an anxiogenic effect in rodents and humans (88, 89). However, it is worth mentioning that high doses of CNP (up to 5 μg), were used in these experiments; at low doses (100 ng), CNP reduced anxiety-like behavior in rats (87). At doses similar to those used for ANP, CNP increased the levels of anxiety-related behavior when administered i.c.v. in rats (88). This effect was abolished by a CRH antagonist, pointing toward an HPA-axis related mechanism (89). In humans, pretreatment with CNP enhanced the emotional effect of the anxiogenic agent CCK-4, and increased the release of ACTH following this treatment (90). CNP was also shown to stimulate cortisol and prolactin release (91). All these finding indicate that CNP is a potent anxiogenic substance that acts by stimulating the HPA-axis. It is therefore that CNP antagonists were considered in anti-anxiety therapy (83).

Atrial natriuretic peptide may modulate alcohol withdrawal-related anxiety. In alcohol-dependant patients, abrupt cessation of alcohol consumption is accompanied by an array of symptoms known as alcohol withdrawal (92). ANP is involved in some of the neurobehavioral aspects of alcohol withdrawal, including anxiety and alcohol craving (93). In mice, i.p. ANP administration attenuated anxiety-like behavior following alcohol withdrawal (94). Handling-induced convulsions resulting from withdrawal were reduced by i.c.v. infusion of ANP, whereas anti-ANP antibodies had the opposite effect (95). Consistently, NPR-A knockout mice showed increased stress-related alcohol consumption and aggravated withdrawal symptoms (96). In humans, acute alcohol consumption elevated plasma ANP levels in healthy individuals (97). In patients with alcohol-dependence, plasma ANP levels were lower during detoxification compared with those in non-drinking individuals (93). The lower levels correlated with alterations in promoter DNA methylation, which was significantly reduced as compared with that in healthy controls (98). On the emotional level, lower ANP levels were associated with increased anxiety and alcohol craving during withdrawal (93, 99). It was postulated that the mechanism of ANP involvement in withdrawal-related stress is mediated through the HPA-axis (100). However, although the HPA-axis stress response affects the patient’s recovery from alcohol addiction (101) as well as relapse rate (102), cortisol and ACTH levels do not correlate with those factors affected by ANP, such as alcohol craving (102) and perceived stress (99). ANP involvement in alcohol dependence is supported by recent genetic studies. A genome-wide association study (GWAS) revealed alcohol dependence to be associated with a single-nucleotide polymorphism located in gene GATA4, which encodes a transcription factor regulating ANP (103, 104). This finding was confirmed by a candidate association study, which found GATA4 to be linked to alcohol dependence at the gene level (105). This genetic variation in GATA4 was also shown to be associated with an increased relapse rate in patients (106), and greater reactivity in the amygdala to alcohol-related images, as shown by functional MRI (107). These results suggest that NP, possibly by modifying the stress response of the HPA-axis, are involved in the pathological states of anxiety disorders and alcohol dependence.

### Future challenges

As described above, there is evidence for the involvement of NP in several brain functions. These observations open exciting new venues for research and drug development. However, many open questions remain to be clarified. In all the cases described above, it appears that NP do not regulate brain functions directly, but rather modulate other endocrine pathways, such as ANG II in BP regulation, or the HPA-axis in anxiety-related disorders. As for their neuroprotective qualities, those could be mediated via other mechanisms activated by brain injury, such as the immune system. The intricate interactions between NP and other cellular systems need to be studied in depth. On the more basic level, although the control of NP biosynthesis and release in the periphery is well established, not much is known about locally produced NP in non-cardiac tissues, the brain in particular. Information is lacking as to the specific cell types in the brain that produce NP, the factors regulating NP production and release, the modes of their elimination, and the neuronal signaling pathways that they affect. Electrophysiological studies are necessary to establish the effects of NP on neuronal excitation and channel activation. It is possible that NP do not directly regulate neuronal activity, but rather modulate it via their effect on glial cells. Studies on the NP effect on calcium release and neurotransmitters uptake in glial cells may help elucidate this point. Also, the interactions between NP and known neurotransmitters and their receptors may be of importance, and should be addressed.

## Guanylin and Uroguanylin

Dietary sodium leads to increased natriuresis in an aldosterone-independent manner. This observation led to the postulation that an additional NH is released from the intestine in response to salt intake (108). Such a hormone was discovered in 1992 – the previously unknown endogenous ligand of the intestinal receptor GC-C, activated by bacterial enterotoxins (109), and termed guanylin. Guanylin was purified from rat jejunum, and it was shown to activate GC-C in T84 human intestinal cells (109). One year later, a second endogenous ligand of GC-C was purified from the urine and intestinal mucosa of opossums, and named uroguanylin (110). More recently, additional members of the family, such as lymphoguanylin and renoguanylin were identified (111, 112). GP are expressed as pre-pro-hormones and are proteolytically cleaved to produce the biologically active peptides (113). They share a similar structure two pairs of cysteine residues forming disulfide bonds in conserved positions that are essential for their biological function (114, 115). Like NP, guanylin and uroguanylin bind to a single-membrane-spanning receptor GC (116). GC-C has a similar GC-C domain architecture to GC-A and GC-B and elicit an increase in cellular cGMP (8), which may account for the similar function of the two peptide families. GC-C is mainly expressed in the intestine, but GC-C transcripts were also found in the adrenal gland, kidney, lung, reproductive system, lymphatic organs, and brain (117, 118).

### Physiological function of GP

Guanylin peptides are produced in the intestine after oral sodium intake and are secreted into the intestinal lumen (119). The resulting increase in enterocyte cGMP stimulates chloride and bicarbonate secretion and inhibits sodium absorption, causing greater secretion of fluids into the intestine (113). Guanylin and uroguanylin also exert long-term effects on the intestine by regulating intestinal cell proliferation (120). In the kidney, these peptides cause increased natriuresis, kaliuresis, and diuresis without changes in GFR or renal blood flow (121). The renal effects of GP are maintained in GC-C null mice (122), suggesting the existence of an additional receptor whose identity is yet to be discovered. Indeed, novel members of the receptor GC family were described in specific cell types in the olfactory system (123, 124).

### GP in the brain

There are few reports on the expression of GP in the brain (118). Their main effect in the CNS is likely endocrine: guanylin and uroguanylin are secreted from the gut and enter the circulation, mainly as prohormones (125, 126), and subsequently affect extra-intestinal tissues such as the kidney (127) and the brain (128). GC-C, the main intestinal receptor for GP, was found in the midbrain (129) and the hypothalamus (128).

#### Uroguanylin in satiety control

The intestine is an important endocrine organ, secreting hormones that centrally regulate satiety and food intake (130). The intestinal hormones are vigorously studied as possible therapeutic targets for the growing public health concern regarding obesity and metabolic diseases (131). Valentino and colleagues identified uroguanylin as a novel satiety hormone and showed that food intake causes increased intestinal prouroguanylin secretion in fasting individuals and mice (128). Administration of bacterial enterotoxins (a GC-C agonist) i.v. or i.c.v. (but not orally) reduced food intake in fasting mice, and i.v. administration of anti-prouroguanylin antibodies blocked this response (128). The receptor GC-C is expressed in the mouse hypothalamus. However, uroguanylin expression was not found in this region, suggesting an endocrine rather than a paracrine mode of regulation (128). To strengthen this postulation, Valentino showed an increase in cGMP in the hypothalamus in response to treatment with prouroguanylin, suggesting that this prohormone is cleaved in the hypothalamus by an unknown enzyme to produce the active peptide form (128). Mice lacking GC-C exhibited impaired satiety that resulted in increased food intake, obesity, and metabolic syndrome. In these animals, as opposed to the normal controls, i.v. administration of bacterial enterotoxins did not reduce food intake (128). Although further validation of this new endocrine pathway is necessary, the study provides strong evidence that uroguanylin is a central mediator of food intake, and it may provide a new therapeutic target for obesity and metabolic diseases (132).

#### GP in behavior

Guanylate cyclase C is expressed in dopaminergic neurons in the midbrain, and GC-C knockout was associated with behavioral changes in mice (129). Gong and collogues showed the expression of the GC-C protein in the ventral tegmental area and substantia nigra compacta in mice (129). Voltage clamp recordings from mouse dopaminergic neurons revealed that guanylin and uroguanylin significantly increased the neuronal activation evoked by metabotropic and muscarinic receptor agonists. This effect was abolished by blocking PKG signaling downstream from GC-C, and it was absent in GC-C knockout mice (129). The knockout mice exhibited increased locomotor activity, high levels of novelty seeking behavior and impulsivity. Such behavior was attenuated by low doses (1 mg/kg) of amphetamine, used to treat attention deficit hyperactivity disorder (ADHD) in humans. It is widely accepted that the dopaminergic system in the midbrain is involved in the etiology of ADHD (133). The GC-C knockout mice were described by the authors as a new animal model for ADHD, as they exhibit some of the symptoms related to this condition (129, 134). However, as the behavioral changes described could mimic several human conditions, further validation of the model is needed, and evidence of the involvement of GC-C in ADHD in humans is required. This pathway can provide new therapeutic targets for diseases involving the dopamine system, such as ADHD and schizophrenia.

### Future challenges

Unlike NP and CS, it appears that GP are not synthesized in the brain, but rather arrive via the circulation from the intestine. However, the link between the intestine and the brain is not clear. Which brain-derived factors, if any, induce GP release from the intestine, and what signaling pathways they regulate require further investigation. The humoral or neuronal pathways that mediate the differential endocrine and paracrine effects of GP on remote organs such as the brain and kidney need to be established. Additionally, new members of the GC receptor family have been recently described in specific sensory neurons (135). There is a strong possibility that there are additional receptors which mediate GP functions in specific brain areas.

## Cardiac Steroids

Cardiac steroids are a group of compounds that bind to and inhibit Na^+^, K^+^-ATPase. These compounds, originally discovered in plants and toad skin, have been used for centuries in Eastern and Western medicine to treat cardiac failure (136). Investigation into these substances started with the search for a missing “third factor” in the regulation of sodium homeostasis, as described in the classic work by de Wardener et al. (137). Although the interest in endogenous CS as the “third factor” has subsided with the finding of the NH, these studies paved the way for the recognition of CS in mammalian tissues and circulation. Rat brain extracts were shown to inhibit Na^+^, K^+^-ATPase activity and ouabain binding (138–140). Consequently, ouabain (141, 142), digoxin (143), and several bufadienolides (144–148) were identified in mammalian tissues, urine and plasma. CS are considered to be produced in the adrenal cortex and hypothalamus (149, 150), although their complete synthetic pathway is unknown. CS are subdivided into cardenolides, such as ouabain and digoxin, and bufadienolides, including bufalin and marinobufagenin. All CS have a steroid nucleus with a lactone ring at position C-17, and a hydroxyl group at C-14. The 5-member- and 6-member lactone rings are essential for the biological function of the cardenolides and bufadienolides, respectively (151). The only established receptor for all CS is the catalytic α subunit of the plasma membrane Na^+^, K^+^-ATPase. In addition to the inhibition of the Na^+^, K^+^-ATPase pumping function (152), the binding of CS results in the activation of signaling transduction cascades, including the Src-kinase, the MAP-kinase, and the PKC signaling pathways (153, 154).

### Physiological function of CS

Na^+^, K^+^-ATPase is an essential enzyme expressed in all mammalian cells. CS have widespread effects in different types of cells, including cardiac myocytes, smooth muscle cells, epithelial cells, and neurons (153, 154). CS play important roles in many physiological and pathological processes, among them sodium homeostasis (155), cardiac function (156), BP (157), cell growth (158), and behavior (159). CS form the link between dietary sodium intake and salt-sensitive hypertension (155). As described below, CS regulate BP and hypertension by their effects in the periphery and in the CNS. Given their presence in the brain and CSF, these substances were postulated to act as neurotransmitters or neuromodulators, and they were shown to be involved in psychiatric conditions such as depressive disorders (159). On the cellular level, CS were found to function in cell growth and proliferation (158) as well as in cell migration (160) and they may be associated with the development of cancer (161).

### CS in the brain

Based on their ability to inhibit ouabain binding and Na^+^, K^+^-ATPase activity, CS were identified in bovine hypothalamus (140), rat brain (138), and CSF from humans (162, 163). Immunohistochemical studies of mammalian brains revealed high concentrations of CS in the paraventricular nucleus and the supraoptic nucleus (164). Cultured rat hypothalamic neurons were shown to secrete CS *in vitro* (164, 165), supporting the premise that the hypothalamus is the source of endogenous brain CS. The only established CS receptor, Na^+^, K^+^-ATPase, is expressed throughout the brain. Three isoforms of this enzyme are expressed in the brain: α1, α2, and α3. They display a complex expression pattern: neurons are the principal source of the α3 isoform (166) [although some express α2, especially in the neonate (167)], whereas glial cells predominantly express α2 (168). The α1 isoform is expressed in all cell types, and considered a house keeping protein. The different subunit isoforms vary in their sensitivity to CS and may mediate differential functions of these substances.

#### CS in central regulation of BP

It is widely accepted that excess dietary sodium is an extremely important factor in essential hypertension (169), although the mechanism by which sodium elevates BP is not clear. A large body of evidence links endogenous CS to the regulation of BP and hypertension. In patients with essential hypertension, plasma levels of ouabain and marinobufagenin were increased in about 40%, with a high correlation with BP (170–175). The plasma levels of these substances in hypertensive patients and in rats increased with sodium intake (176–178). Several animal models for hypertension showed increased circulating levels of CS (178–180). Furthermore, prolonged infusion of ouabain produced hypertension in animals (181–183), but had no effect in genetically modified ouabain-insensitive mice (183, 184). In transgenic mice, a greater natriuretic response to sodium loading was demonstrated in animals expressing a highly CS-sensitive Na^+^, K^+^-ATPase α1 subunit (185). Studies on mice carrying mutations in the gene encoding α2 showed that ouabain-induced elevation of BP in rodents was mediated via this isoform: reduction of the expression level of α2 was associated with increased BP (186). In contrast, animals overexpressing α2 were hypotensive (187). Treatment of hypertensive rats with anti-digoxin antibodies (185, 188) or anti-marinobufogenin antibodies (178) administered to rats on a high sodium intake, resulted in a marked reduction in BP. Endogenous ouabain was put forward as a putative target for the treatment of hypertension; the ouabain inhibitor rostafuroxin showed promising results in hypertensive rats (189). Studies by Leenen and colleagues indicated that CS involvement in BP regulation is partially mediated by their effect in the CNS. The first indication of brain involvement came from experiments in SHR, in which adrenalectomy did not prevent the increase in CS levels following high sodium intake (177). Lesions in the most anteroventral part of the third ventricle (AV3V) showed that this region is essential in mediating the pressor effects of increased CSF sodium concentration via endogenous ouabain (190, 191). The effects of both acute and prolonged ouabain infusion in sodium-loaded rodents were abolished by administration of ANG II type 1 receptor blockers such as losartan (192, 193), as well as in transgenic rats with reduced brain renin-angiotensin pathway activity (194). These results pointed to the involvement of this pathway in the effect of ouabain. All of these finding led to a unifying hypothesis regarding the role of CS in sodium-induced hypertension: sodium loading increases the levels of ouabain in salt-sensitive individuals (195, 196). In addition to induction of vasoconstriction in the periphery, ouabain also acts in the brain, where it activates the renin-angiotensin pathway, causing sympathetic activation, vasoconstriction and consequently, an elevation in BP.

#### CS in depressive disorders

Mood disorders include major depression and dysthymia, characterized by depressive episodes, and bipolar disorder (BD) marked by both depressive and manic episodes. These conditions pose a growing public health concern in the Western world. The etiology of these diseases is not completely understood. Early reports of the psychiatric effects of CS came from doctors describing a syndrome termed “foxglove frenzy” or “digitalis delirium” in patients with digitalis intoxication (197). More recently, a comprehensive hypothesis was put forward, linking brain CS levels and Na^+^, K^+^-ATPase activity with BD (198, 199). BD has consistently been associated with abnormalities in Na^+^, K^+^-ATPase activity in erythrocytes (200, 201). A significant mood-related decrease in the enzyme’s activity was found in manic BD patients (202). Furthermore, Na^+^, K^+^-ATPase density was significantly lower in BD patients than in major depressed and schizophrenic patients (159). The plasma levels of endogenous CS were found to be significantly decreased in manic individuals as compared with those in normal controls (203, 204). Conversely, the levels of these compounds were higher in the parietal cortex of BD patients (159). More recently, it was found that there is a reduction in brain Na^+^, K^+^-ATPase α1 isoform expression in mice treated with the mood stabilizer lithium (205). An allelic association between BD and a Na^+^, K^+^-ATPase α subunit gene (ATP1A3) was reported (206). We have demonstrated the prominent linkage to BD of six single-nucleotide polymorphisms (SNPs) in the three genes of the Na^+^, K^+^-ATPase α isoforms. Haplotype analysis of the α2 isoform showed the significant association of two loci haplotypes with BD (207). A genetic knockdown of the neuron-specific Na^+^, K^+^-ATPase α3 isoform induced manic-like behavior in mice (208). Numerous studies have demonstrated that i.c.v. injection of ouabain induces hyperactive behavior in rats (159, 209), which could be ameliorated by administration of mood stabilizing drugs such as lithium (210). Reduction of the endogenous brain CS level by i.c.v. injection of anti-ouabain antibodies had anti-depressive effects in rats (159, 211). This was reflected by significant changes in catecholamine metabolism in the hippocampus and ventral tegmentum, two regions known to be associated with mood disorders (211). The molecular pathway underlying the CS behavioral effect is unknown. Ouabain injected i.c.v. elicited the activation of the ERK and Akt signaling pathways in the brain (212, 213), which are known to be activated via Na^+^, K^+^-ATPase. Other effects of ouabain include a reduction in brain-derived neurotrophic factor (BDNF) (214), activation of mammalian target of rapamycin (mTOR) signaling (213) and an increased level of oxidative stress (215). These findings strongly link the Na^+^, K^+^-ATPase and CS system to the etiology of depressive disorders, and BD in particular, and suggest their potential application in future drug development.

### Future challenges

Despite the identification of cardenolides and bufadienolides in mammalian tissue in many independent studies (see Cardiac steroids), some still question the validity of these findings. Recently, it was claimed that ouabain, the most studied cardenolide, could not be detected in human plasma (216). This issue must be clarified. An additional major missing piece of information for the establishment of CS as neurotransmitters or neuromodulators is the elucidation of their biosynthetic pathway in the adrenal gland and brain. Although the available literature supports the notion that these steroids are synthesized in mammals, the key enzymes involved have not been identified. This issue was recently reviewed in Ref. (217). Several CS were identified in the human body. It was postulated that the different α isoforms of the Na^+^, K^+^-ATPase serve as receptors for the different CS. Which of the CS are involved in brain functions, and which isoform combinations they activate are topics for future research.

## Interactions of ANP with CS and GP

Mutual interactions exist between CS and NP in the periphery and in the brain. Ouabain and digoxin were shown to cause increased ANP expression and secretion in rat and rabbit atria (218–221), and in anesthetized dogs (222). In patients with congestive heart failure, i.v. administration of digitalis increased the plasma levels of ANP and BNP (223). Indeed, ANP regulates the secretion of CS in the brain (224–226). ANP decreased the release of CS from rat brain extract when added to the tissue, or when administered i.v. to the animals prior to their sacrifice (224). On the other hand, another study showed that i.c.v. injection of synthetic ANP increased blood CS levels, whereas i.v. administration or incubation with this peptide had no effect (225). The effect of ANP on CS release was abolished by lesions in the AV3V region (226), the area in the hypothalamus that is thought to mediate CS central regulation of BP (191). In addition to secretion regulation, NP and CS interact at the functional level. ANP differentially modulates the effect of marinobufogenin in the rat heart and kidney (227). Ouabain was shown to antagonize the effect of ANP on vasorelaxation in rabbit aorta and in dogs (228, 229), whereas ANP abolished an ouabain-induced increase in aldosteron secretion (230). Administration of anti-ouabain antibodies caused increased sensitivity to ANP-induced vasodilation in rat aorta (231). In rat heart muscle preparations, ANP was shown to attenuate several effects of ouabain, including ouabain-induced increase in contractility, Na^+^, K^+^-ATPase and ERK phosphorylation (232). ANP also interacts with GP. Santos-Neto and colleagues showed synergism between ANP, guanylin, and uroguanylin in the kidney (233). They demonstrated in an isolated perfused rat kidney that pretreatment with ANP significantly enhanced the natriuretic, kaliuretic, and chloriuretic responses to low doses of guanylin and uroguanylin (233). Low doses of ANP enhanced GP induced diuresis, and vice versa (233). Since GP and NP activate GC receptors, their interaction may be mediated through the shared second messenger, cGMP (6, 116, 234). These initial studies suggest the physiological crosstalk between ANP and CS, particularly in the cardiovascular system and in the brain, and between ANP and GP in the kidney. More studies are needed to deepen our understanding of the nature of these interactions, which may be of significance in the regulation of peripheral and central functions of the NH.

## Conclusion

**Figure 1:**
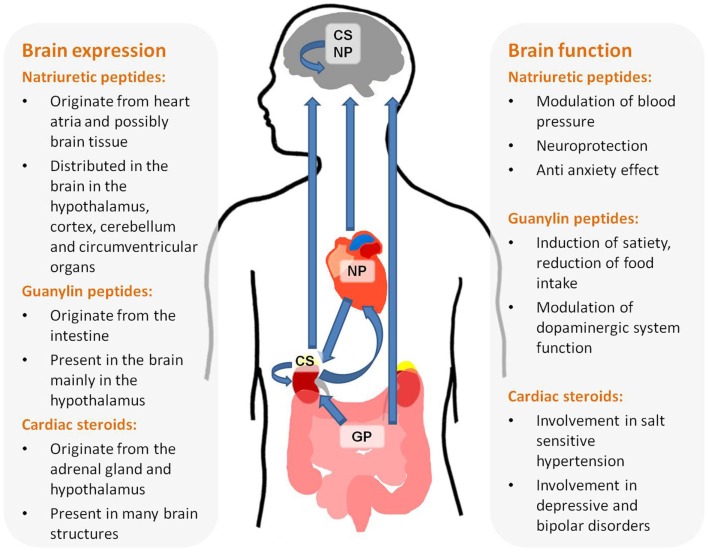
**Origin, brain distribution, and function of natriuretic hormones**.

This review summarizes the available data implicating NH in brain function. There is a vast amount of data supporting the assessment that the three families of NH, NP, Guanylins, and endogenous CS are present in the brain and participate in high brain functions (see Figure 1). These include central regulation of BP, satiety, neuroprotection, and behavior. In depth research of these effects will not only increase our thorough understanding of brain function but may also lead to new treatments for brain-related diseases.

## Conflict of Interest Statement

The authors declare that the research was conducted in the absence of any commercial or financial relationships that could be construed as a potential conflict of interest.
